# Variance partitioning reveals contrasting random effect contributions to the density and species composition of malaria-transmitting mosquitoes in western Burkina Faso

**DOI:** 10.1186/s13071-026-07406-0

**Published:** 2026-04-18

**Authors:** Tin-Yu J. Hui, Patric Stephane Epopa, Abdoul Azize Millogo, Franck A. Yao, Dao Koulmaga, Florian Noulin, Abdoulaye Diabate, Austin Burt

**Affiliations:** 1 Department of Life Sciences, Silwood Park Campus, Imperial College London, Ascot, UK; 2 Institut de Recherche en Sciences de la Santé/Centre Muraz, Bobo-Dioulasso, Burkina Faso; 3 Institut Des Sciences Des Sociétés, 03 BP 7047 Ouaga 03, Ouagadougou, Burkina Faso; 4 Department of Molecular Biology and Genetics, Aarhus University, Universitetsbyen 81, 8000 Aarhus C, Denmark

**Keywords:** *Anopheles gambiae*, Malaria, Vector density, Species composition

## Abstract

**Background:**

Spatial–temporal variation exists in the density and species composition of malaria-carrying mosquitoes, which will in turn influence the transmission of the disease. While there has been extensive research on seasonality and other main drivers of the vector populations, the heterogeneity partitioned as random effects at various spatial–temporal scales is just as important but has not attracted the same attention.

**Methods:**

To investigate the relative contributions of the between-house, between-village and between-year variations, as well as other house-level covariates such as inhabitant number and bed net usage on vector density and species composition, intensive pyrethroid spray catches (PSC) sampling was conducted across a 60-month period between 2012 and 2019 from four villages in the Sudano-Sahelian region of Burkina Faso.

**Results:**

For density, measured by female *Anopheles gambiae s.l.* counts, our modelling showed that the between-house variation was the largest variance component, followed by the between-year then between-village variation, after accounting for seasonality and other covariates. Density increased with the number of inhabitants within a household but was uncorrelated with bed net presence. A subset of female mosquitoes was genotyped for species identification, and the composition of *An. coluzzii* and *An. gambiae*, the two dominant vectors in the region, varied markedly across villages without an overall trend. The between-village variance contributed up to 76% of the total random variation in species composition, followed by the between-year variance. The between-house variation was statistically insignificant. Neither household size nor bed net usage had any impact on species composition.

**Conclusions:**

Interestingly, the between-house component of variation was the largest contributor when measuring mosquito density, but it was the least important for species composition. For between-village variation, the converse was found. Together with the baseline entomological data, the variance components help parameterise potential field trials for novel vector control programmes and monitoring.

**Graphical Abstract:**

**Figure:**
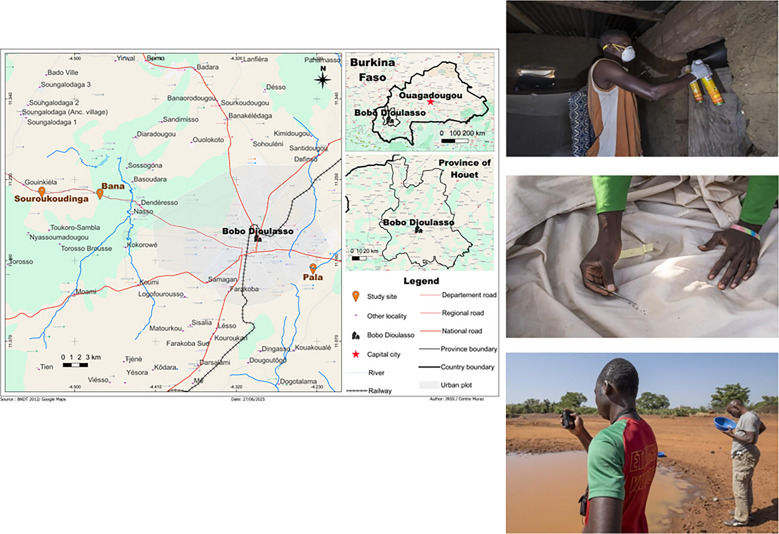

## Background

Despite the continued effort and investment in malaria control programmes, regions in sub-Saharan Africa remain disproportionally affected by this vector-borne disease. Harrowing figures of 263 million cases and over half a million deaths are still being reported on the continent, accounting for about 95% of the global malaria burden [1]. Recent modelling predicted that the current intensity and range of interventions (such as long-lasting insecticidal nets and indoor residual spraying) can at best maintain the status quo but are unable to drive malaria towards eradication [2]. In fact, the recent increase in malaria incidence coincides with the vectors’ resistance to insecticides [3, 4], highlighting the need for developing complementary cost-effective and sustainable novel technologies for malaria control. One example is the sterile insect technique (SIT), which has previously been implemented by the release of radiation-sterilised individuals across multiple insect species that are deemed harmful [5]. SIT can also be achieved via genetic modification (GM) of the vectors, such as by engineering a transgene into the males of *Aedes aegypti* for dengue control [6]. Beyond SIT, there has been extensive theoretical and laboratory-based research on developing gene drive systems for *Anopheles gambiae s.l*., a species complex whose members contain the primary malaria vectors in sub-Saharan Africa. Gene drive allows the transgene to be passed on to offspring and beyond in a super-Mendelian manner to achieve longer-term population suppression or replacement [7, 8].

For contagious or vector-borne diseases, the basic reproductive number ${R}_{0}$ is the most important metric to quantify transmission, with ${R}_{0}<1$ being a requirement for eradication. In malaria, ${R}_{0}$ is defined as the expected number of persons to be infected one generation after the malaria parasite was introduced in a naïve population, or equivalently the number of secondary cases in a population without previous exposure to the disease [9]. As the life cycle of the malaria parasite consists of both mosquito and human phases, complex models are deployed to characterise the host–parasite–vector interaction [10]. The entomological inoculation rate (EIR), for example, connects the sporozoite rate (i.e. the prevalence of the parasite in the salivary glands of female mosquitoes), and human biting rate (HBR) per night, which has the mosquito density per person embedded [11]. Under the classical models ${R}_{0}$ and vectorial capacity are functions of these entomological rates at equilibrium. To reduce the intensity of transmission, most vector control programmes aim to suppress factors concerning vector density or competence [9].

Malaria transmission is never uniform across space and time. Previous estimates of ${R}_{0}$ varied hugely from ~ 1 to > 1000 across Africa [9], and mosquito density fluctuates across seasons, often by orders of magnitude [12]. In fact, heterogeneity exists on almost all levels: from mosquito density and species distribution across countries, mosquito exposure in households from the same village, down to the variable susceptibility to malaria infection of individuals. While earlier works have focussed on identifying the main drivers (such as environmental and climatic factors) associated with the trends in malaria vectors and cases [13], it is equally important to examine the different portions of random variation. For example, the between-house, between-village and between-year variations all contribute to the total unexplained variation, but their relative contribution may vary. Through the extensive mosquito collection survey this study has the following aims: (1) to estimate the above variance components concerning mosquito density and species composition at after accounting for seasonality and other fixed covariates; (2) to discuss how these random effects impact vector density and malaria transmission; (3) on the basis of the relative strengths of the random effects, to provide recommendations on the potential designs and considerations for clustered randomised controlled trials (cRCTs) under various intervention scenarios.

## Methods

### Mosquito sampling

**Fig. 1:**
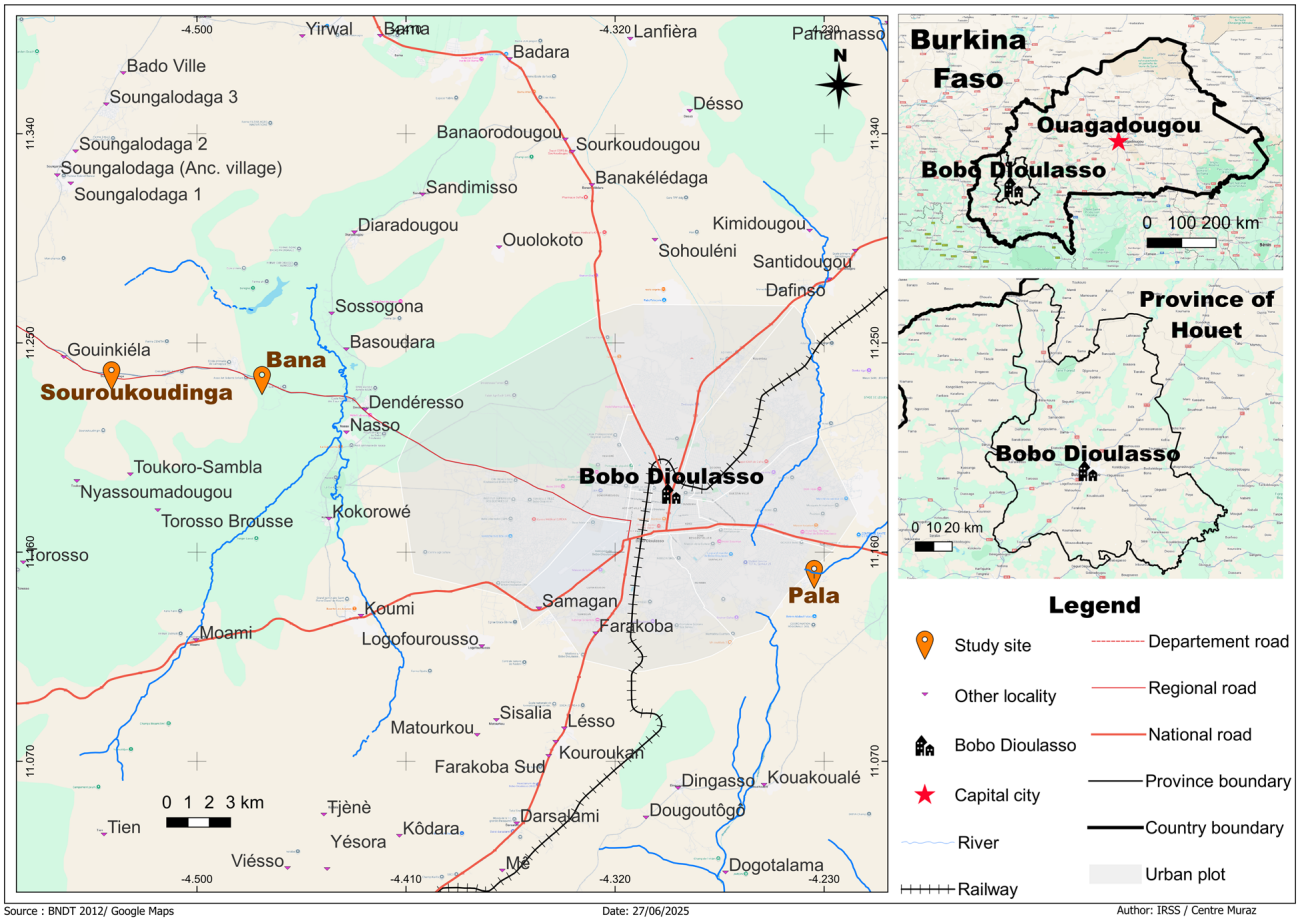
Location of our sampling locations in the Houet Province of western Burkina Faso. The coordinates of the sampling locations are: Bana [11.233, −4.472], Souroukoudingan [11.235, −4.537], Pala [11.151, −4.234]. In the centre of the map is Bobo-Dioulasso, the second largest city in Burkina Faso, where our research base locates. There are two sampling locations from Bana: Bana Village and Bana Market, which are about 23 km from Bobo-Dioulasso. Souroukoudingan is about 7 km further away in the same direction. Pala is on the opposite direction approximately 6 km from Bobo-Dioulasso

**Fig. 2:**
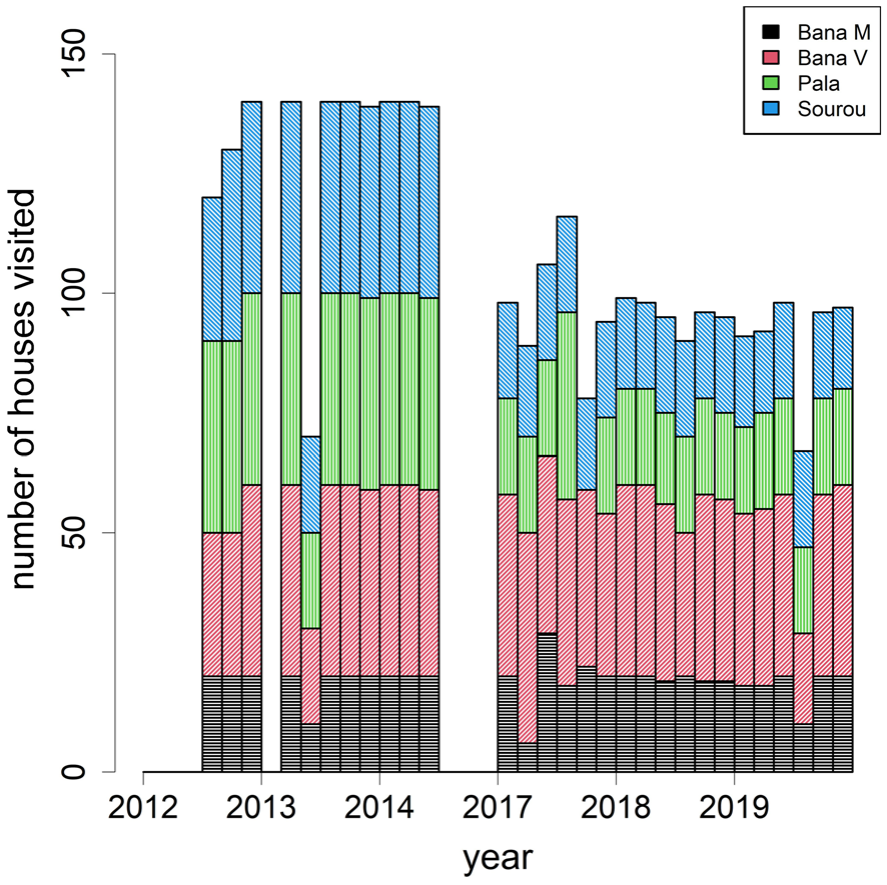
Sampling effort per village over time (total of 3133 visits, excluding those with missing data). Each bar represents the number of houses visited for PSC collection per two months. Sampling was paused from July 2015 to Dec 2016

Mosquitoes were sampled in the houses from four villages in Burkina Faso’s Sudano-Sahelian: Bana Market, Bana Village, Soukroudingan and Pala (Fig. 1), where *An. gambiae* (s.s.), *An. coluzzii* and *An. arabiensis*, the sibling species of the *An. gambiae s.l.*, are the primary vectors. The first three villages are located to the west of Bobo-Dioulasso, where our research base situates, while Pala is on the opposite direction from the rest [14]. Bana’s two collection sites, Bana Market and Bana Village, are separated by a small semi-permanent river with different human activities [15]. Two phases of collection were conducted: from July 2012 to June 2014 and throughout 2017–2019, covering a total of 60 months. Monthly collection was scheduled aiming to sample 20 houses per village per month. Houses were chosen randomly from a pool of known houses on the basis of local information, from which half of the houses were repeatedly visited over time (fixed houses), while the remaining half were further randomised each month (random houses). For each village, we attempted to sample all houses in the same or consecutive days to minimise the effect of potential confounding factors. For Pala and Souroukoudingan, the sampling interval switched to once every two months in the second phase (Fig. 2). Sampling took place after sunrise via indoor pyrethroid spray catches (PSC) in the bedroom (or the main living area if there was an open-plan living space), using a well-known and locally market available insecticide spray Kaltox^®^ Paalga (Sphyto Company, Burkina Faso). All mosquitoes were morphologically identified in the field [16]. The targeted organisms *An. gambiae s.l.* were preserved in 80% ethanol for further analysis. During the second phase of collection polymerase chain reaction (PCR) was performed on a subset of the preserved samples for species identification, such that the composition of the species (within *An. gambiae s.l.*) per visit was also known. The genotyping was based on the detection of the SINE 200× locus [17].

### Statistical analysis

Each house visit generated a line of record, including the number of female *An. gambiae s.l.* from the PSC collection, the date of collection and a unique house ID. Other potential covariates concerning the visit were also measured by our team members, such as the number of residents, and whether bed net was in use. Female *An. gambiae* s.l. per visit was the main response to proxy indoor mosquito density. To model seasonality, we used a six-level factor variable “month–pair”, with two consecutive months being pooled as one (e.g. January and February as one level, and so on) for a more balanced design. A Poisson generalised linear mixed model (GLMM) was fitted. Seasonality, household size and the use of bed nets (yes/no) were treated as fixed effects. Overdispersion was included by introducing an observation-level random effect [18]. Villages, year of sampling and unique house IDs were considered as random effects. The interaction between village and seasonality was also incorporated for additional variability. For species composition, a similar binomial GLMM was fitted to the proportion between the female counts of the two most dominant species in the region. Analyses were conducted in R version 4.3.1 [19] with packages lme4 and emmeans [20].

## Results

**Fig. 3:**
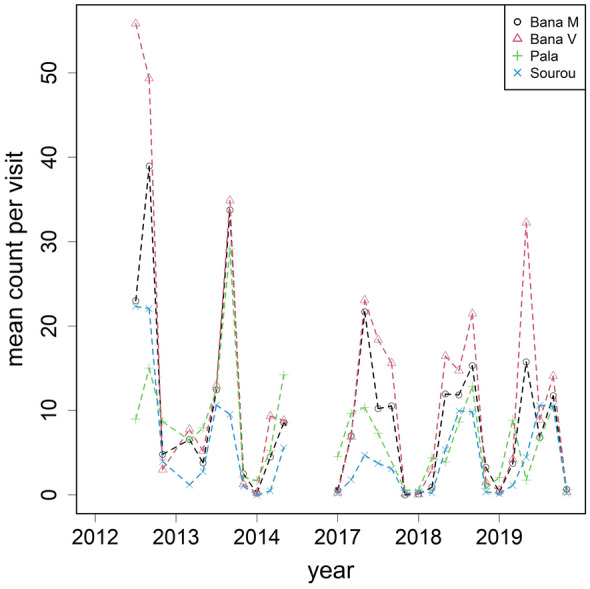
Mean female *An. gambiae* s.l. collected per visit by village over time. Note that sampling was paused after June 2014 then resumed at the beginning of 2017

We endeavoured to follow closely the intended sampling plan, with 3212 visits made. After removing entries with missing information, 3133 visits from 383 distinct houses were retained over the sampling period (Fig. 2). Mosquito density as measured by the number of female *An. gambiae s.l.* is summarised in Fig. 3: A total of 28,919 female *An. gambiae s.l.* were collected, with an average of 9.2 per visit. The high mean count came with huge variation across visits ranging from 0 to 333 per visit. The mean household size was just over 3 persons—this value was subsequently used to classify household size into categories (none, small with 1–3 persons, large with > 3 persons). Bed nets were installed in about 90% of the visits.

A Poisson GLMM was fitted to the counts (Table 1). Seasonality was well defined within a calendar year with a period of low and high counts. Lower average counts were observed between November and February, then increased when transitioning into the next season towards the peak at the months of September and October. There was about a 30-fold difference between the lowest and highest mean counts. As we proxied seasonality with the month–pair variable of six levels, we performed contrasts to test whether their mean counts were different (Table 3). All pairwise comparisons were significant, apart from the three levels of months between May and October where they shared the same mean. The presence of bed nets reduced mosquito count by 14%, but the effect was not statistically significant (*P* = 0.099). Count increased with household size: compared to an empty house a threefold (*P* = 0.013) increase was observed if there were ≤ 3 occupants, then a further 53% increase (*P* < 0.001) for a bigger household with > 3 occupants. For the random components (Table 2), overdispersion, modelled through an observation-level random effect, accounted for the largest share of the unexplained variance in the model (65%). The second largest term was the between-house variation (14%). The village–seasonality interaction accounted for an additional portion of the variance (8%), indicating that seasonal patterns differed somewhat between villages. The between-village variance was found to be the lowest at 4.6%. All five variance components were statistically larger than zero (Table 3).

**Table 1:** Fixed effect estimates from the Poisson GLMM on the counts of female *An. gambiae* s.l.

Fixed effect	*df*	Comparison(s)	Estimate (s.e.)	*P*-value
Month–pair (seasonality)	5			0.000***
		(see Table 3)		
Bed net	1			
		No versus Yes	-0.15072 (0.091)	0.099
Household size	2			0.000***
		None versus Small	1.110 (0.446)	0.013*
		Small versus Large	0.427 (0.066)	< 0.001***
		None versus Large	1.537 (0.449)	0.000***

The significance of the fixed effects and *P*-values was based on likelihood ratio tests (LRTs), while Wald *P*-values were computed for pairwise comparisons. Effect estimates are on log-scale. Asterisks are given to indicate statistical significance: (***) *P* < 0.001, (**) *P* < 0.01, (*) *P* < 0.05

**Table 2:** Random effect estimates and percentage contributions from the same Poisson GLMM, ranking from the largest. LRTs were performed to obtain the *P*-values

Random effect	Number of levels	Variance estimate	Percentage	*P*-value
Overdispersion	3133	1.4413	64.88	0.000***
Between-house	383	0.3127	14.07	0.000***
Between-year	6	0.1881	8.47	0.000***
Between-village x Seasonality	24	0.1768	7.96	0.000***
Between-village	4	0.1025	4.61	0.048*

**Table 3:** Pairwise contrast for the seasonality effect in the Poisson GLMM (Table 1)

	0102	0304	0506	0708	0910	1112
0102		0.000***	0.000***	0.000***	0.000***	0.000***
0304	2.151		< 0.001***	0.003**	0.000***	0.000***
0506	3.212	1.061		0.638	0.285	0.000***
0708	3.066	0.915	−0.146		0.130	0.000***
0910	3.544	1.394	0.332	0.479		0.000***
1112	0.798	−1.353	−2.414	−2.268	−2.746	

Seasonality was categorised into six factor levels. For example, “0102” refers to the counts of January and February combined, “0304” for March and April, and so on. Contrasts (lower-triangular) and *P*-values (upper-triangular) were computed via the R package “emmeans”. Note that these are raw *P*-values before adjusting for multiple comparisons

**Fig. 4:**
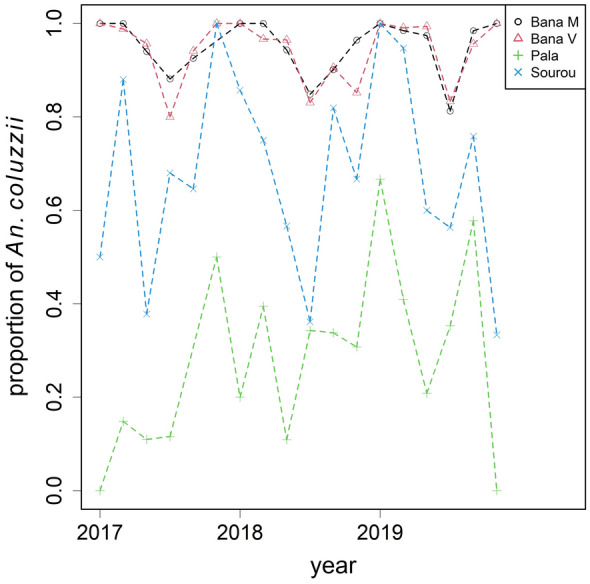
The relative proportion of *An. coluzzii* and *An. gambiae* by village over time. Species identification was performed to a subset of mosquitoes collected during 2017–2019

**Fig. 5:**
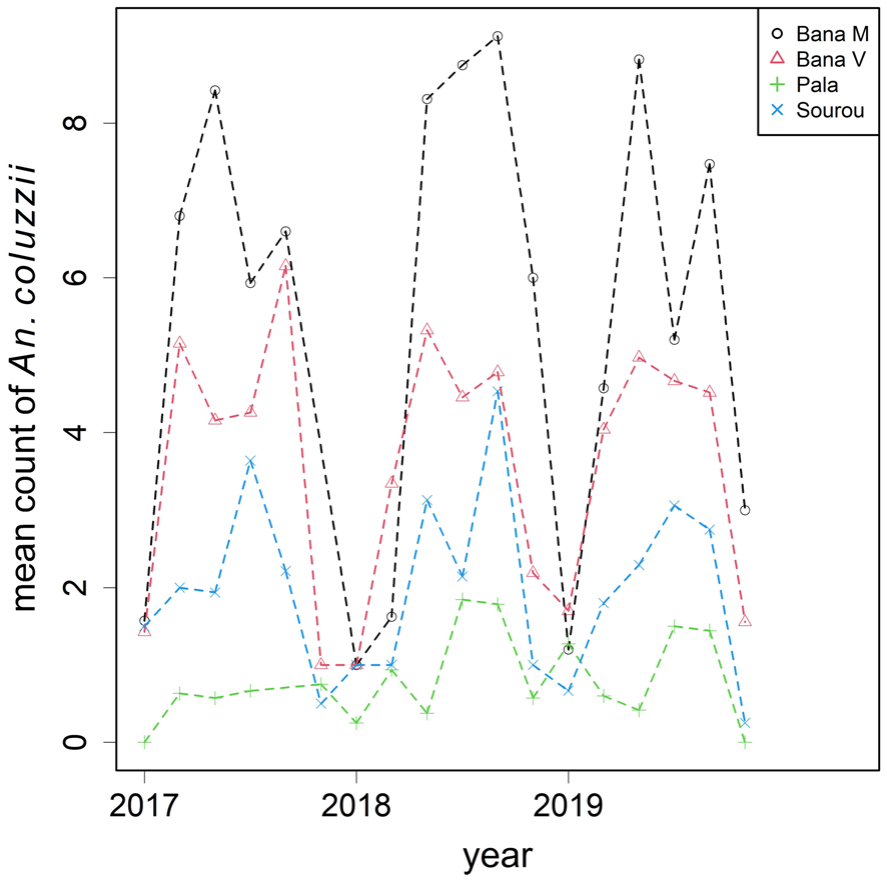
Mean number of female *An. coluzzii* per house visit by village over time

**Fig. 6:**
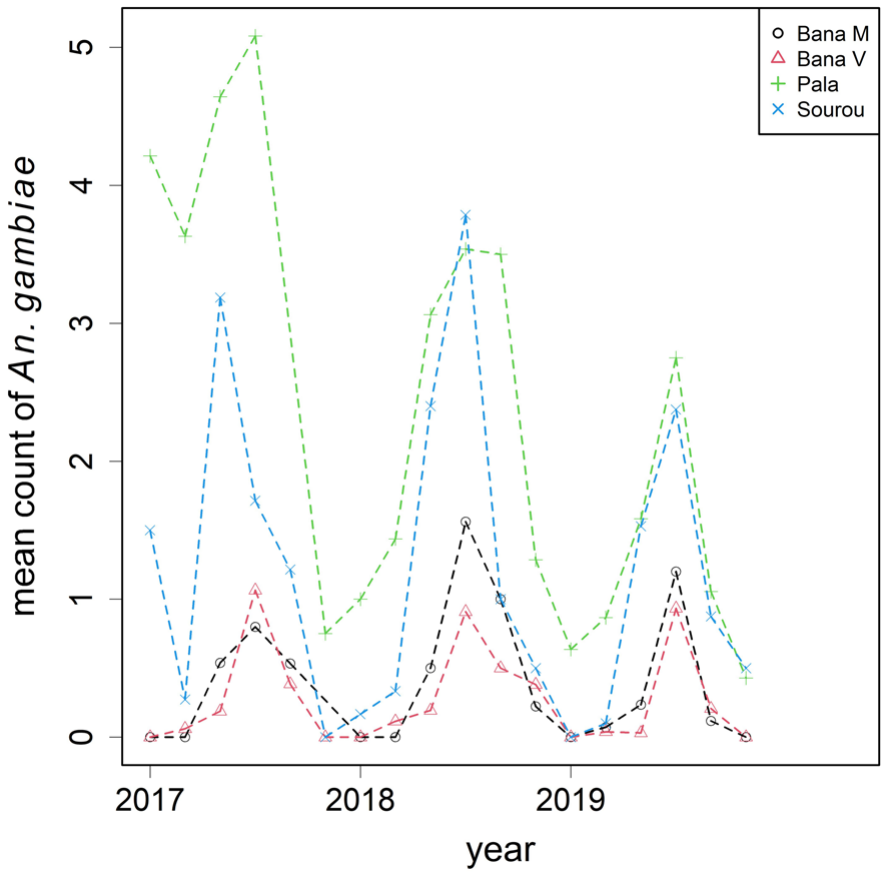
Mean number of female *An. gambiae* per house visit by village over time

A subset of female *An. gambiae s.l.* from the second phase of collection (2017–2019) was genotyped for species identification. After genotyping, 3626 were identified as *An. coluzzii* and 1102 as *An. gambiae*, yielding an overall ratio of approximately 3:1 from 985 house visits (Figs. 5, 6). The monthly aggregates are summarised in Figs. 4–6. Besides the two dominant species, a small proportion of *An. arabiensis* (~ 5%) was also found but excluded from subsequent statistical analyses. In Bana Market and Bana Village, *An. coluzzii* was the dominant species making up over 90% of the samples, and similarly 60% in Souroukoudingan, while in Pala it was the minority (24%). Although species composition varied throughout a year, the four villages did not seem to follow the same dynamics nor a pre-defined pattern within a year. A binomial GLMM was then fitted to model the relative proportion of the two species. For the fixed effects, neither the use of bed nets (*P* = 0.77) nor household size (*P* = 0.89) had an influence on the proportion (Table 4). For the random components (Table 5), the between-village variation was estimated to be the largest, explaining 77% of the total random variance. This was followed by overdispersion (11.4%) and the village-seasonality interaction (3.9%). The between-house variation was not significant (1.5%, *P* = 0.26). In other words, setting overdispersion aside, the relative importance of the random components were the exact opposite of those for mosquito density. Model outputs and diagnostic checks (e.g. residual deviance plots) are available on github.

**Table 4:** Fixed effect estimates from the binomial GLMM on the proportion between female *An. coluzzii* and *An. gambiae*, with *P*-values obtained from LRTs

Fixed effect	*df*	*P*-value
Month-pair (Seasonality)	5	0.028*
Bed net	1	0.771
Household size	2	0.886

Effects are reported on logit scale

**Table 5:** Random effect estimates and percentage contributions from the binomial GLMM on species proportion, ranking from the largest

Random effect	Number of levels	Variance estimate	Percentage	*P*-value
Between-village	4	3.476	76.47	0.000***
Overdispersion	985	0.520	11.43	0.000***
Between-village × seasonality	24	0.304	6.68	0.000***
Between-year	3	0.179	3.92	0.000***
Between-house	200	0.0678	1.49	0.263

LRTs were performed to obtain the *P*-values

## Discussion

Several baseline collections have been conducted in Burkina Faso and beyond to evaluate vector density over space and time [21–23]. Our results concur with existing knowledge that mosquito density (as measured by female counts in this study) exhibits strong seasonal trends. The study region is characterised by a tropical climate marked by two main seasons, with average annual temperatures between 25 and 30 °C and rainfall of 800–1100 mm [24]. The period at which we consistently observed high mosquito counts agreed with the broad definition of the rainy season in the region, which usually defined as the months from June to September, with May and October being in transition [15] The peak at the late rainy season near October was also detected by Dao et al. [12] in a Sahelian village in the neighbouring country of Mali. After reaching the peak, a period of low counts immediately followed from November into February. The clean switch between the two seasons is a signature of the Sahelian region as the surface water disappears, drastically reducing the availability of larval sites [25, 26]. In parallel to our census size estimates, genetic estimates of effective population size showed similar patterns of seasonal variation [27].

While many existing collections aimed to correlate seasonality to other deterministic drivers with mosquito dynamics through monthly summaries, or to identify biological processes behind them, curating individual PSC records gives the opportunity to analyse covariates on finer scales such as at the observational and household level. We expect our results to hold for most PSC collections, although different sampling methods allow for the collection of different types of information about the vectors. Bed net use, a factor considered as a fixed effect in this study, was associated with a 14% reduction in mosquito density (but not significant) despite a high coverage of over 90%. Airborne pyrethroids, albeit less volatile than some other insecticides, can inhibit mosquitoes from feeding even they can detect human odours, suggesting some degree of spatial repellency [28, 29]. However, whether the mere presence of pyrethroid treated bed net can suppress indoor mosquito density remains largely unknown. There has been an increase in frequency of insecticide resistance variants linked to pyrethroid (such as 1014F) during the same period, which can potentially explain its relatively low repelling ability [3]. We found a strong relationship between density and household size, a factor which was previously thought to be less important [21]. Another dimension of this work is to estimate the random effects, which quantify the heterogeneity that has not been accounted for by our main effects. We intentionally fitted a 6-level month–pair variable for seasonality with 24 additional random village–seasonality interaction terms to our GLMMs to saturate the seasonality effect, allowing us to focus on the remaining random components. Note that part of the seasonality can be accounted for by abiotic factors, such as rainfall, as demonstrated previously. The relative magnitude of the variance components provides an indication of heterogeneity across different spatial and temporal scales, although these components should not be interpreted as direct causal drivers of mosquito density. Several random components on various levels were considered: Overdispersion, assigned to each visit, was also the largest component on mosquito density. It includes the intrinsic variation of indoor mosquito density, sampling method, and potentially other abiotic or idiosyncratic sources of heterogeneity that have not been accounted for the existing fixed and random effects in the model. By incorporating household-level information, the between-house variance was estimated to be the second largest component, indicating some houses were more susceptible to mosquitoes even though they were exposed to similar conditions as their neighbours. The difference could be due to building material, usage, and proximity to larval sites. Although it is impossible to suggest an exhaustive list of covariates affecting mosquito density, further modelling, including the use of geographic information system (GIS) data, is warranted to dissect this variance component [21]. This suggested an uneven distribution of mosquito contact and potential exposure to biting across households, providing an indicative measure of the heterogeneity of population-level transmission metrics such as ${R}_{0}$. The between-village and between-year variances were minor with respect to density, each contributing to only a few percent of the total random variation.

There is no surprise that the two primary vectors, *An. coluzzii* and *An. gambiae*, were abundant in the region. Our binomial GLMM concluded that the proportion of the two species was unaffected by bed net usage or household size. The four sampled villages tended to have their own underlying proportions and fluctuations over time: Souroukoudingan and the two sites from Bana were predominantly *An. coluzzii*, while Pala was the opposite. There were no consistent patterns in species proportions nor observable switch between the two seasons. The large between-village and village–seasonality interaction illustrated how heterogeneous species composition can be across a landscape. It is known that the two species coexist yet have contrasting preferences. *An. coluzzii* prefers sites with permanent freshwater while *An. gambiae* can tolerate intermittent access to water [25]. Bana and Souroukoudingan have access to water year-round owing to agricultural activities which explains the strong presence of *An. coluzzii*. Pala, in contrast, is a peri-urban village dominated by trade and handicrafts [22]. The species’ persistence mechanisms may also affect the relative abundance in the dry season and transition months. It is generally thought that *An. coluzzii* aestivates locally during the dry season, thus an immediate population expansion is expected when the aestivators reemerge, whereas *An. gambiae* takes days to respond to the arrival of the rainy season as immigrants from further afield recolonise the focal population [30]. In short, the large between-village and lack of between-house variance were the key signatures of species composition. In fact, the relative importance of the random components in species composition is the exact opposite of that of density (Table 6), which itself is a striking finding. Future work should include the monitoring of *An. arabiensis*, which was also present in our study, especially in urban settings where it appears to be colonising [31].

**Table 6:** Descriptive summary of the fixed and random factors affecting mosquito density and species composition, based on the numerical results from the two GLMMs reported in Tables 1–5

Factor	Density	Species composition
Seasonality	Clear seasonal pattern with a rainy and dry season per year	No clear rainy and dry season patterns. Villages tend to have their own seasonality
Bed net installation	Not significant	Not significant
Household size	Density increased with household size	Not significant
Between-house variance	Large	Not significant
Between-year variance	Small but non-zero	Small but non-zero
Village–seasonality interaction variance	Moderate	Moderate
Between-village variance	Small but non-zero	Large

For the random effects, the relative importance is reported

Both mosquito density and malaria transmission are subject to strong spatial–temporal variation. Besides our findings on density, a previous survey in the same region estimated a 25-fold increase in EIR from the dry to rainy season, mainly driven by the sharp rise in human landing rate [22]. Both malaria incidence and mosquito density found their peaks towards the late rainy season in Burkina Faso [32], meaning seasonal control strategies may be required [33], further straining the healthcare system. Seasonality may also affect the overall efficacy of GM vector control and the optimal schedule of releases [34, 35]. While the heterogeneity in transmission across seasons or climatic zones have been anticipated, the unexplained variance components impose additional challenges on the road to the elimination of malaria. Persistent transmission can be maintained even under continued and effective malaria control [36]. It only requires a minority of human individuals with dry season exposure to constitute an infectious reservoir [37]. The higher the random variances in mosquito density, the higher the chance these small pockets of space are created for permanent transmission. Classical epidemiological models show that, with heterogeneous biting, a factor of $(1+\alpha )$ is applied to ${R}_{0}$ where $\alpha$ is the squared coefficient of variation (CV) in biting rate [9]. In other words, ${R}_{0}$ increases linearly with the variance in biting rate if all else is equal. While biting rates are best estimated from human landing catches, our PSC collection and between-house variance estimate in density could be carefully extrapolated to inform the degree of heterogeneity linking to indoor exposure and biting. With the many layers of random variations, the compounding effect on ${R}_{0}$ could potentially be significant even if the mean estimate suggests otherwise. Biting can also occur outdoors, thus measuring the outdoor mosquito density and species composition with other trapping methods would complement this study [38]. In terms of reporting, increased spatial and temporal heterogeneity in malaria cases in low transmission settings reduces the usefulness of national or regional level trends in incidence or prevalence [39].

Any new intervention needs to undergo phases of rigorous testing and trial. A cRCT is the gold-standard study design for evaluating public health programmes, where interventions are addressed to some clusters then compared with those without [40, 41]. Our dataset and parameter estimates contribute towards baseline data collection for future field trials with entomological endpoints and inform some key considerations concerning trial design. Many mosquito intervention programmes must be addressed to a cluster (e.g. a village of households) as a whole, hence it is the basic unit for randomisation. In the traditional parallel design, clusters will be assigned to either control or intervention throughout without cross-over. If an intervention targets the entire species complex leading to an overall decrease in mosquito density, then the reduction in courts in the clusters receiving interventions compared with the control is the main outcome measure as the entomological endpoint. The intervention effect can be evaluated by a Poisson GLMM like the one we fitted to the baseline count but with an additional binary variable indicating those receiving intervention [42]. The coefficient of the indicator variable estimates the intervention effect of log(1−*G*), where *G* is the intended suppression level. Some novel vector control strategies can be tailored to target a subset of vector species while keeping the rest intact, and thus the corresponding outcome measure is the ratio between the targeted and non-targeted species. Proportional data are then fitted to a binomial GLMM to estimate the intervention effect, which is again log(1−*G*) on the logit scale if the intervention is designed to suppress the population of the targeted species by *G*. Sampling should focus the rainy season to maximise mosquito collection per unit effort: large baseline counts in the rainy season provide headroom for suppression as well as a lower CV. Species composition can also be measured with greater precision as the binomial variance is inversely proportional to sample size. In addition, observation-level covariates can also inform the randomisation of clusters, such as in restricted randomisation, or even in a paired design to further improve statistical power and balance [43].

The random effects also play an important role in the parameterisation of cRCTs through the intracluster correlation coefficient (ICC), which is the ratio of the between-cluster variation over the sum of the within- and between-cluster variations. On the basis of our random effect estimates (Tables 2, 5), for the density measure, the individual variances (i.e. overdispersion and between-house variation) dominated the total variation, and thus the ICC is expected to be small, which helps reduce the number of clusters required. We recognised that the four study villages were chosen on the basis of logistical considerations as they are in the close vicinity (~ 30 km radius) of Bobo-Dioulasso. A full-scale cRCT is likely to be conducted on a regional or national scale with many more clusters, and the between-village variance is expected to expand with geographical coverage. For species composition between *An. coluzzii* and *An. gambiae*, owing to the low between-house variance, the ICC is much higher such that the effective sample size approaches the number of clusters. On a positive note, the homogeneity among houses within the same village has the potential to simplify recruitment as a few households could represent the entire cluster. Owing to the difference in their ICCs, it is unlikely that the same trial design will perform equally in the monitoring of mosquito density and species composition.

Another aspect that affects density and species composition is temporal variation. Some (a few percent) between-year variation was detected throughout the three to six years of baseline collection. Temporal variation is less of a problem for the classical parallel cRCT design as comparisons are made within the same period between the two arms. A stepped-wedged design, in which all clusters are in control condition initially and then start receiving the intervention gradually, may be confounded by this effect [44]. This design on the other hand may benefit scenarios with high ICC, as all clusters with have both pre- and post-intervention stages for comparison [44].

## Conclusions

This study provides valuable baseline data for future entomological trials. We partitioned the unexplained heterogeneity into random effects of various spatial–temporal scales, which in turn characterise the persistent transmission of malaria and further inform ICC in cRCT designs. Depending on the intervention, the outcome measure can either be the density of the species complex or the proportion between the targeted and un-targeted ones. With the many combinations of scenarios, further studies are necessary to fully explore their complex interplay, particularly on the quantitative aspects of trial design such as power and sample size determination.

## Data Availability

The full dataset and computing notebooks are available here: https://github.com/tinyuhui/vector_composition.
